# Sex-Related Differences in Chronic Pain: A Narrative Review by a Multidisciplinary Task Force

**DOI:** 10.3390/medicina61071172

**Published:** 2025-06-28

**Authors:** Maurizio Marchesini, Diego Fornasari, Silvia Natoli, Elena Vegni, Arturo Cuomo

**Affiliations:** 1 Department of Anesthesia, Critical Care and Pain Medicine, Mater Olbia Hospital, 07026 Olbia, Italy; 2 Department of Medical Biotechnology and Translational Medicine, Università Degli Studi di Milano, 20133 Milan, Italy; diego.fornasari@unimi.it; 3 Department of Clinical-Surgical Diagnostic and Pediatric Sciences, University of Pavia, 27100 Pavia, Italy; silvia.natoli@unipv.it; 4 Unit of Pain Therapy Service, Foundation Istituto di Ricovero e Cura a Carattere Scientifico (IRCCS), Policlinico San Matteo, 27100 Pavia, Italy; 5 Clinical Psychology, Department of Health Sciences, University of Milan, 20122 Milan, Italy; elena.vegni@unimi.it; 6 Unit of Clinical Psychology, Santi Paolo and Carlo Hospital, 20142 Milan, Italy; 7 Division of Anesthesia and Pain Medicine, Istituto Nazionale Tumori, IRCCS—Fondazione G. Pascale, 80131 Naples, Italy; a.cuomo@istitutotumori.na.it

**Keywords:** chronic pain, sex-difference, pain factors, biological factors, psychosocial factors, socioeconomic factors, chronic pain therapy

## Abstract

*Background and Objectives:* Chronic pain (CP), defined as pain persisting for over 3 months, is a significant cause of global disability and affects more than 20% of individuals in Western countries, including Italy. Substantial evidence indicates a higher prevalence of CP among women, who also experience greater sensitivity, increased frequency, and a longer duration of pain. The impact of CP on quality of life, daily functioning, and employment is profound, particularly for women. However, chronic pain research has insufficiently addressed sex and gender differences, contributing to suboptimal and inequitable care. This neglect limits the development of personalized treatment strategies and, when combined with an aging population and women’s longer life expectancy, highlights an increasing societal and economic burden. *Materials and Methods:* The authors conducted a narrative review of studies examining biological, psychological, or social determinants of sex-related differences in CP perception or treatment. Each thematic area was reviewed by at least two authors, who critically appraised the literature. Their analyses were refined through iterative group discussions to develop concise, evidence-informed recommendations for personalized and equitable pain management. *Results:* Sex differences in CP arise from a range of factors, including biological mechanisms such as hormonal and genetic influences, psycho-social factors such as depression and anxiety, and socio-economic determinants, such as income and education levels. These factors also affect sex-specific outcomes of analgesic treatments currently available. Identifying these risk factors and tailoring treatment strategies to sex differences can significantly improve CP management. Such a personalized approach is essential for advancing precision medicine in CP management. Even in the absence of molecular or genomic biomarkers, adopting a biopsychosocial model that considers sex and gender differences, symptoms, physiological indicators, medical history, lifestyle, and psychological aspects may substantially enhance patient outcomes. *Conclusions:* This review provides a comprehensive analysis of sex differences in CP perception, stressing the importance of individualized, interdisciplinary approaches in pain management. Addressing both the biological and psycho-social contributors to pain in men and women is critical for guiding healthcare professionals in implementing precision pain medicine strategies, ultimately fostering more equitable and effective care.

## 1. Introduction

The International Association for the Study of Pain (IASP) describes pain as an “unpleasant sensory and emotional experience associated with, or resembling that associated with actual or potential tissue damage” [1,2].

Globally, CP is a major cause of disability [3], affecting over 20% of the population [4,5,6]. Numerous studies consistently report that CP is more prevalent among women [7,8,9,10,11,12,13,14,15]. Two recent surveys evaluated the prevalence of CP in Italy: the Censis-Grünenthal survey [16] and the Italian National Institute of Health survey [17]. According to these surveys, CP affects 19.7% of adults with moderate-to-severe CP [16] and 24.1% overall [17]. The Italian National Institute of Health further analyzed sex-based differences, reporting a prevalence of 19.7% in men and 28.1% in women, with disparities increasing with age [17].

CP substantially impacts on quality of life (QoL), daily functioning, and employment [13]. Despite this, nearly half of individuals with CP do not receive adequate pain management, even though they attend regular healthcare visits, with women disproportionately affected [18,19,20,21]. Research indicates that women report greater pain intensity [15,22], longer pain duration [13], and heightened pain sensitivity [23,24,25] and employ different coping strategies compared with men [23]. These disparities influence satisfaction with pain relief from medication [18,20,21]. Given the aging population and women’s longer life expectancy, the societal and economic impact of CP is expected to grow [26,27].

Despite increasing evidence, both research and clinical practice have historically overlooked sex as a critical variable in pain mechanisms and management. Preclinical studies have predominantly relied on male models, while clinical trials frequently lack sex-disaggregated data. This persistent gap has resulted in treatment strategies that do not adequately consider the specific needs of women and men with CP, potentially leading to suboptimal outcomes.

The growing focus on precision medicine and the biopsychosocial model of care calls for a more nuanced understanding of how sex and gender differences affect chronic pain. Addressing these differences is essential to improving equity in care and personalizing therapeutic interventions.

The heightened prevalence of CP in women is attributed to several factors [15], including biological factors: hormonal fluctuations [28]; chronic overlapping conditions [29]; age [17,30]; neuroimmune interactions [31,32,33]; sex differences in pharmacodynamics; pharmacokinetics [34] and response to opioid receptor antagonists [35,36,37,38]; psycho-social factors including gender biases [39,40], cultural roles [41,42], and andronormativity [23]; and factors related to socio-economic status (SES), including income, education, and access to healthcare [43,44,45,46].

These determinants are reflected in population-level data. For instance, the Censis-Grünenthal survey in Italy found that the leading causes of moderate-to-severe CP differ by sex: osteoarthritis, migraines, osteoporosis, and headaches are most common in women, while men are more frequently affected by vascular diseases, poor circulation, post-operative pain, and neuropathies [16].

Socio-demographic and psychological factors further influence sex differences in CP [47]. Recognizing this, the IASP established a working group in 2007 to explore how social constructs, cultural roles, behaviors, and relationships shape CP prevalence among men and women.

This narrative review, developed by a multidisciplinary expert task force, aims to

- Summarize current evidence on sex-related differences in chronic pain epidemiology, mechanisms, and treatment response;
- Identify existing research gaps and barriers in clinical practice;
- Provide expert-driven suggestions for implementing more personalized and equitable approaches in chronic pain management.

By integrating biological, psychological, and social perspectives, this review intends to support clinicians and policymakers in developing sex-aware, multidisciplinary pain care strategies that reflect the complex and individualized nature of CP.

## 2. Materials and Methods

The authors, Italian experts in pharmacology, psychology, and pain medicine, performed a narrative review of the literature. The literature search was conducted using PubMed/MEDLINE and Google Scholar databases. The search terms used are specified in Table 1.

No restrictions were applied to the study design or publication date in order to ensure a comprehensive analysis.

Full-text articles were retrieved when relevance was unclear or the abstract matched the inclusion scope. Reference lists of selected articles were manually examined to identify additional relevant publications. Articles were included if they addressed any biological, psychological, or social determinant of sex-related differences in CP perception or treatment.

Exclusion criteria were (a) non-English-language publications, (b) studies focusing exclusively on acute or cancer-related pain, (c) articles without explicit reference to sex or gender variables, and (d) editorials or commentaries without original data or review syntheses.

Each thematic domain (e.g., epidemiology, biological mechanisms, psychological factors, pharmacological approaches) was assigned to at least a couple of specialist authors who critically appraised the selected literature in that domain.

Clinical domain: M.M and A.C.

Preclinical and pharmacological domains: D.F. and S.N.

Psychological domain: E.V. and M.M.

The resulting analyses were then discussed within the full author group through iterative rounds of collaborative evaluation. This process allowed for a consensus on key findings and expert-driven suggestions based on both literature evidence and clinical experience.

## 3. Results and Discussion

### 3.1. Sex Differences in Chronic Pain: Prevalence, Perception, and Underlying Factors

Historically, research on sex differences in CP has been limited, particularly in preclinical studies, despite women constituting the majority of CP sufferers. A review of articles published in Pain found that more than 75% of studies included exclusively male subjects [22]. A systematic analysis by Beery et al. [48] across multiple disciplines confirmed this male bias, with the most pronounced disparities observed in neuroscience (5.5:1), pharmacology (5:1), and physiology (3.7:1).

One reason for this disparity was the outdated assumption that the female estrous cycle and hormonal fluctuations would introduce excessive variability [157]. However, neuroscience studies in rodents have debunked this assumption, demonstrating that females are not inherently more variable than males in terms of behavior or traits [49,50]. Despite this evidence, assessing the variability of the menstrual cycle in human pain research remains challenging. Ideally, evaluations should be conducted during the same menstrual cycle phase, as hormone fluctuations may influence pain perception [28]. Simple methods, such as home-based urine or saliva tests to predict ovulation or track the date of the last menstruation, could be easily incorporated into clinical research to account for these fluctuations [51].

In 2016, the National Institutes of Health (NIH) introduced the “Sex as a Biological Variable” policy, requiring NIH-funded research to consider sex differences [158]. The policy increased the inclusion of women in clinical studies, highlighting the importance of understanding sex-based differences in pain processing [52], which is crucial for identifying risk factors and developing personalized treatments.

Globally, CP affects more women than men [7,8,9,10,11,12,13,14,15], although prevalence rates vary by country [4,53]. An international survey of 17 countries reported CP in 45% of women compared with 31% of men [54]. In Italy, sex differences in CP began to emerge in the 35–44 age group, where 14.2% of women and 10.8% of men reported CP [17]. This gap widened with age [17]. Among individuals aged 65–74 years, 41% of women and 28.6% of men reported CP, while in those aged 84 and older, the prevalence rose to 63.1% in women and 49.6% in men, reflecting a nearly 14-percentage-point difference [17].

Additionally, CP conditions often coexist within the same individual, and women are disproportionately burdened by overlapping pain disorders, such as migraines, fibromyalgia, low back pain, and osteoarthritis [15,29,55,56]. Biological and psycho-social factors, including hormonal influences and cultural roles, may contribute to these disparities [15].

Men and women also differ in their approach to pain management. Studies suggest that women tend to use a variety of coping strategies, such as emotional and cognitive techniques [23,24], while men are more likely to perceive pain as a threat to masculinity, often resorting to denial or avoidance [23].

In Italy, 18.9% of women and 16.7% of men rate pharmacological treatments for moderate-to-severe pain as highly effective [16]. Treatment satisfaction varies with age [17]. Younger men (aged 18–34 years) report higher satisfaction with pain therapies than women in the same age group, but this gap narrows with age. Women aged 65–74 express slightly higher satisfaction [17]. However, in women over 75, satisfaction declines significantly, with only 12% finding treatments effective, compared with 18% of men in the same age group [17]. Partial relief is more common in older patients (77% of women vs. 68% of men) [17].

The panel of experts suggests that research efforts should adopt standardized methodologies and balanced sampling by sex, avoiding over-reliance on male models. Clinical assessments should consider both biological and sociocultural influences on reported symptoms.

### 3.2. Biological and Psycho-Social Factors Could Drive Sex Differences in Chronic Pain

**Figure 1:**
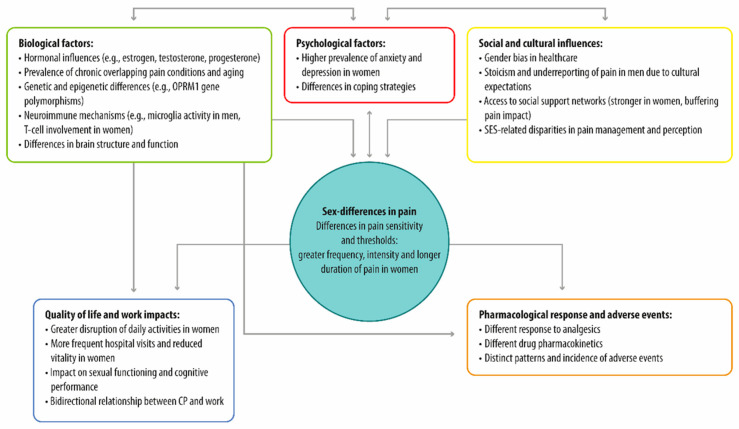
Multifactorial determinants of sex differences in pain, organized into interconnected clusters.

Building on the recognition of the existence of sex differences in pain, understanding the underlying biological and psycho-social factors becomes essential. These factors do not operate in isolation but interact in complex ways, influencing pain experiences and treatment responses (Figure 1; Table 2) [159].

#### 3.2.1. Biological Factors

Hormonal fluctuations are major biological contributors to sex differences in pain perception [28].

Pain sensitivity to heat and pressure tends to increase during ovulation when progesterone levels peak [28]. In contrast, testosterone has demonstrated anti-nociceptive effects, potentially explaining the lower prevalence of certain CP conditions in men [57].

Common causes of CP more prevalent in women include musculoskeletal conditions such as rheumatoid arthritis [58], osteoarthritis [59], and fibromyalgia [60], as well as migraines [61], tension headaches [62], low back pain [63], temporomandibular joint disorders [64], complex regional pain syndrome [65], burning mouth syndrome [66], neuropathic pain [67], and chronic pelvic pain [68]. In Italy, low-back pain is the most frequently reported site of moderate-to-severe CP in both sexes, affecting 29.9% of women and 31.0% of men [16]. However, women more frequently report moderate-to-severe pain in the knees, head, feet, and hips, whereas men report higher rates of moderate-to-severe neck pain [16].

Conditions such as endometriosis [69] and interstitial cystitis [70], which affect the reproductive and urinary systems, are major contributors to chronic pelvic pain. Endometriosis alone affects approximately 10% of women of reproductive age [69]. In contrast, CP conditions that occur more frequently in men, such as cluster headaches, are less common in women [15,71].

Age is another important factor in CP, with older adults being more likely to experience noxious stimuli or injuries that trigger CP [30]. The Italian National Institute of Health reports that women aged 45 years and older, particularly those with multiple health conditions, report CP more frequently than men [17]. In the absence of multimorbidity, the disadvantage for women is statistically significant only among those over the age of 75 years. In contrast, in terms of pain intensity, this disadvantage emerges as early as the age of 65 years [17].

With aging, pain-related conditions become more difficult to diagnose and treat, especially in the presence of cognitive decline or dementia [72]. Interestingly, pain characteristics such as duration, severity, and number of pain sites are stronger predictors of ongoing pain in older women than men [73]. Recent studies also suggest that CP may accelerate the subjective experience of aging, making older individuals feel older than their biological age [74].

Neuroimmune interactions play a pivotal role in sex differences in pain. In men, microglia, the immune cells of the brain, are central to neuropathic pain mechanisms [31,32,33], whereas in women, T cells and other immune cells play a more active role in modulating pain sensitivity [31,32,33].

This suggests that treatments targeting microglial activity may be more effective for men, while therapies focusing on T-cell regulation may benefit women. Interestingly, some research challenges this distinction, with studies showing that microglia can influence pain sensitivity in both sexes under certain conditions [32]. For instance, genetic manipulation of microglia has been shown to reverse pain hypersensitivity even in female rodents [75,76,77]. Further complexity arises from the microglial modulation of pain through various pathways, including synaptic transmission [32].

The panel of experts suggests that future studies should identify sex-specific biomarkers and molecular targets, ensuring that translational research accounts for biological diversity in treatment development (Table 2).

#### 3.2.2. Psycho-Social Factors

Historically, sex, defined by biological attributes [78], and gender, encompassing socially constructed roles, behaviors, expressions, and identities [78], have often been conflated in research, leading to misinterpretations. A more nuanced understanding of sex as a continuum is essential, especially when considering variations, such as intersex individuals or atypical chromosomal patterns (e.g., XXY, XXXY), which may influence different pain experiences [79].

Gender biases in healthcare further complicate the recognition and treatment of pain, particularly in conditions traditionally associated with one sex over the other [80]. For example, women often experience delayed diagnoses and inadequate pain management for conditions like spondyloarthritis, myocardial infarction, and cardiogenic shock, where they receive fewer diagnostic tests and less attention than men [81,82,83,84].

Women’s reports of pain are frequently overlooked or attributed to emotional causes, reinforcing stereotypes that they exaggerate symptoms [39,80]. This gender bias affects healthcare providers, who may perceive women as more emotional and less resilient to pain, leading to inadequate treatment [39,40]. Conversely, men are often portrayed as stoic, expected to endure pain silently or take assertive action, while women are considered more vulnerable and expressive [23,85].

Cultural expectations about gender roles significantly influence pain responses. Experimental studies show that individuals scoring high in masculine traits display greater pain tolerance, while those with higher femininity scores report increased pain sensitivity [41,42]. Interestingly, when both men and women are given the same pain tolerance expectations in controlled settings, gender differences in pain perception and tolerance tend to disappear [42], highlighting the strong impact of social conditioning.

The medical field also exhibits andronormativity, where male norms dominate clinical practice, often leading to the marginalization of women’s pain experiences, making them less recognized and insufficiently addressed in medical practice [23].

Lower SES is consistently associated with greater pain severity, disability, and reduced access to effective care [43,86,87,88]. These disparities are especially pronounced among women, who face compounded biases related to both gender and class [43,44]. Stereotypes portraying low-SES women as less perceptive or passive in response to pain contribute to under-treatment, whereas higher-SES women are often seen as more competent in managing pain, revealing systemic biases in clinical assessment and care delivery [45,46].

CP also imposes a disproportionate financial burden, with direct costs exceeding those of other major disease categories [89].

Employment is another key domain affected by CP: prevalence is markedly higher among unemployed individuals [90], and women are more likely to adjust or leave employment due to pain [91,92,93,94].

Coping strategies and mental health also differ between the sexes, influencing pain experiences. Women are more likely to use maladaptive coping mechanisms, such as catastrophizing [95], increasing the risk of CP [24,96]. They also tend to be more open in talking about pain and more likely to seek medical care, as evidenced by their greater attendance at pain clinics than men [97]. In contrast, cultural expectations about masculinity often lead men to underreport pain, resulting in untreated conditions and disparities in pain management [97]. For instance, the Censis-Grünenthal survey found that women were more likely to seek help for CP management compared to men (42% vs. 33.4%) [16].

CP has a bidirectional relationship with mental health [98], with conditions such as anxiety and depression both contributing to and resulting from CP [99,100,101]. The Italian National Institute of Health has found that women with CP generally have lower Mental Health Index (MHI) scores than men (63.2 vs. 68.6), a disparity observed at all ages and education levels [17]. Notably, MHI scores decline more abruptly as pain intensity increases, with an earlier onset in women (around age 35) compared with men (around age 55). Depression is highly prevalent in CP patients, with a reported prevalence of 10–50% [6,102,103,104], and occurs more frequently among women [17,102]. Studies also indicate a positive correlation between depression and CP [105].

Anxiety also disproportionately affects women with CP across all age groups and education levels [17], further exacerbating the burden of CP on their daily lives and relationships [6,106].

Women with CP report worse QoL outcomes than men, including greater impairments in physical, emotional, and social functioning [107,110]. Based on the recent surveys mentioned, women with moderate-to-severe CP report lower vitality levels (48.9 vs. 55.3) and greater difficulty with daily activities [16], particularly as pain intensity increases [17]. Among older adults, women are disproportionately affected, with 54.2% experiencing motor difficulties and 30% facing severe self-care challenges, compared with 38.2% and 21% of men, respectively [17].

The severity of depressive symptoms is strongly associated with the extent to which CP interferes with daily activities. The Italian National Institute of Health reports that CP impacts daily functioning in 74.2% of individuals with severe depressive symptoms, 58.8% with moderate symptoms, and 11.7% without depression [17]. Although data are not stratified by sex, it is reasonable to infer that higher depression rates among women with CP make them more vulnerable to disruptions in daily life.

CP also impacts sexual functioning [109] and cognitive performance, with women being more frequently affected, especially those with comorbid conditions such as type 2 diabetes and neuropathic pain [110]. Social support plays a critical role in mitigating the impact of CP, but its availability and quality vary by sex and cultural context. Women generally report stronger support networks [111,112], which can buffer the negative effects of CP. Conversely, younger men with CP often lack such support due to cultural norms of stoicism, leading to greater difficulties in self-care and daily activities.

Interestingly, Swedish research has shown that women entering pain rehabilitation programs report higher levels of pain acceptance and activity engagement than men despite experiencing similar pain levels [113]. Notably, in this study, women had a mean age of 45 years, whereas men had a mean age of 51 years [113].

The panel of experts suggests that pain management strategies must consider sex-specific hormonal and immune contributions, alongside gender-influenced coping behaviors and treatment-seeking attitudes.

### 3.3. Pharmacological Approaches to Chronic Pain Management

#### 3.3.1. Sex Differences in Chronic Pain Treatment Response

Women exhibit distinct responses to analgesics, influenced by genetic, hormonal, and pharmacokinetic factors [34]. Sex differences in ibuprofen efficacy have been investigated across various pain models, with mixed results across studies. One study suggests that men may be more sensitive to ibuprofen in certain pain models, such as sunburn-induced pain, where it was more effective at lowering skin temperature in men [114]. A sex difference was also reported in electrically induced pain, with ibuprofen-treated men exhibiting greater pain tolerance than women [115,116].

However, not all studies support this distinction. A third-molar extraction pain model found no significant sex differences in the analgesic response to ibuprofen [117]. Similarly, in endodontic pain, ibuprofen was equally effective in both sexes, while pentazocine/naloxone demonstrated greater analgesic efficacy in women than in men [118]. These discrepancies highlight variability in study outcomes, which may be attributed to differences in experimental design, pain stimulus type, and sample size.

Whether sex differences in pain treatment response extend to opioid therapy remains uncertain. While pentazocine appears to have greater analgesic efficacy in women in the oral surgery pain model, this difference is not observed in other experimental pain models, including heat, pressure, and ischemic pain [119]. Animal studies suggest that males generally exhibit greater activation of μ-opioid receptors (MOR) [123], which may partially explain the sex-related differences in opioid efficacy [121]. Estradiol, a key female hormone, has been shown to reduce MOR activity, further complicating opioid response in women [122,123].

Conversely, a recent narrative review suggests that mixed µ-k-opioid agonist-antagonists and pure µ-agonists tend to be somewhat more effective in women based on opioid consumption patterns, particularly after a few days of administration [124]. Data from patient-controlled analgesia (PCA) indicate that female patients self-administer significantly less morphine than men [124]. Furthermore, experimental PCA studies have observed greater morphine-induced analgesia in women [125]. In contrast, women require higher morphine doses for post-operative pain relief [37,38].

Sex-specific responses to opioid treatment are influenced by both genetic and epigenetic factors. A key genetic contributor is the A118G polymorphism in the opioid receptor Mu 1 (*OPRM1*) gene, which alters opioid efficacy across sexes [126]; individuals carrying the 118G allele require higher opioid doses for adequate analgesia [127]. In women, epigenetic modifications, such as the methylation of *OPRM1* and catechol-O-methyltransferase (*COMT*) genes, have been associated with reduced opioid efficacy [126], suggesting that epigenetic modifications may contribute to sex-related disparities in pain management.

The panel of experts suggests that pain management strategies must also consider sex-specific genetic and epigenetic contributions (Table 2).

#### 3.3.2. Sex Differences in Adverse Drug Reactions and Pharmacokinetics in Chronic Pain Management

Women are well-documented to experience a higher incidence of adverse effects (AEs) from medications than men [128]. Research indicates that women are approximately 30% more likely to experience AEs, resulting in nearly twice as many AEs as men [128]. Among patients treated for CP, a notable 79% of women report AEs [129]. This disparity persists even with commonly prescribed medications, such as non-steroidal anti-inflammatory drugs (NSAIDs) [130].

The underlying causes of sex differences in AEs are likely multifactorial, with pharmacokinetic differences [131] playing a significant role. Women tend to have slower gastric motility, longer intestinal transit time, and higher gastric pH [132,133,134,135], all of which can affect the absorption and bioavailability of certain medications and lead to slower clearance than men [136]. Moreover, women’s higher body fat percentage alters the volume of distribution for lipophilic drugs like opioids, potentially affecting clearance and bioavailability [137].

In an observational study examining sex differences in the analgesic response to oxycodone/naloxone (OXN) and tapentadol compared to other opioids in patients with chronic non-cancer pain, women exhibited lower tolerability to these treatments and required hospital care more frequently. This trend was particularly pronounced in the OXN group, where women required higher doses than men to achieve pain relief [138]. The pattern of adverse events also varied by sex [138]. Regarding differences among opioid groups, patients receiving OXN experienced the highest incidence of AEs per patient, with a particularly pronounced effect in females [138]. Among women in the OXN group, constipation was the most frequently reported AE, affecting 71% of patients, significantly higher than the prevalence observed in women treated with tapentadol or other opioids (*p* < 0.01) [138]. Additionally, 48% of women receiving OXN reported weight changes, a rate significantly higher than that observed in male patients (*p* < 0.05) [138].

Conversely, men across all opioid groups reported a higher prevalence of sexual AEs than women, with significantly elevated rates of sexual impotence (9–20%) and loss of libido (10–15%) [138]. Notably, men treated with OXN exhibited a higher incidence of sexual impotence than those in other opioid groups [138]. This finding aligns with previous research demonstrating that OXN significantly reduces testosterone levels (*p* = 0.004), whereas tapentadol does not affect testosterone concentrations in male patients (≤64 years old) with severe chronic low back pain and normal baseline hormone levels before treatment [139].

A key pharmacokinetic parameter is drug metabolism. Women exhibit greater activity of phase I biotransformation enzymes, particularly CYP3A4 and CYP2D6, which are crucial for opioid metabolism [132].

The CYP2D6 enzyme is especially important for converting codeine and tramadol into their active metabolites [140]. A study by Lopes et al. [140] showed a pattern across CYP2D6 phenotypes, from poor to ultra-rapid and rapid, where women faced a significantly higher risk of adverse reactions and a lower risk of poor pain control. This trend was not observed in men [140]. Interestingly, even among normal and intermediate metabolizers, women exhibited nearly twice the rate of AEs as men, reinforcing the idea that sex is a critical factor in determining both the effectiveness and safety of opioids metabolized by CYP2D6 [140].

Additionally, men tend to have higher renal clearance of certain medications due to higher glomerular filtration rates and tubular secretion [141,142], which affects the elimination of drugs such as pregabalin [143].

Women are significantly more likely to stockpile medications (68% vs. 48% of men) and use adjuvant therapies for pain management (39% vs. 20% of men) [144]. This increased medication use may raise the likelihood of drug-to-drug interactions and intensify side effects [145].

Additionally, pain in women is more frequently associated with psychological factors, making them more likely than men to be prescribed anxiolytic or antidepressant medications [39]. This further increases the risk of drug interactions and contributes to a less favorable drug tolerability profile in women.

Analgesic drugs can significantly impact women’s reproductive health. In particular, opioid medications can impair endocrine function and disrupt sex steroid balance [146], leading to hypogonadism, amenorrhea, oligomenorrhea, and reduced libido [147,148].

In the obstetrical population, opioids can have far-reaching consequences for both maternal and neonatal health [147,149]. Opioid exposure during pregnancy is associated with increased neonatal morbidity and mortality, including conditions such as neonatal abstinence syndrome, which affects infants born to opioid-exposed mothers [150]. Furthermore, opioid use in premenopausal and menopausal women has been linked to reduced hormone levels, including dehydroepiandrosterone, estradiol, and luteinizing hormone [147], underscoring opioids’ significant impact on endocrine function and reproductive health.

Corticosteroids, another commonly used class of drugs for pain and inflammation, also have the potential to disrupt the hypothalamic–pituitary–adrenal (HPA) axis [151]. The HPA axis plays a crucial role in stress regulation and hormonal balance, and glucocorticoid receptors are widely expressed throughout the female reproductive system [151]. Long-term corticosteroid therapy may lead to altered reproductive function, potentially causing menstrual irregularities and fertility issues in women [151]. Understanding how both opioids and corticosteroids interfere with hormonal regulation is essential for optimizing long-term care in women with CP.

The panel of experts suggests that clinicians should apply sex-aware prescribing practices, particularly for opioids and NSAIDs, and advocate for the inclusion of sex-disaggregated data in clinical trials.

#### 3.3.3. Psychological Interventions in Chronic Pain: Enhancing Quality of Life Through Tailored Therapies and a Multidisciplinary Approach

While pharmacotherapy is central to CP management, it often fails to address the complex emotional, social, and psychological aspects of the condition [152]. Psychotherapy becomes invaluable in this context. Although it does not directly eliminate pain, it can significantly improve physical, emotional, social, and occupational functioning, offering a holistic approach to CP management. By fostering better coping mechanisms, these therapies can enhance QoL despite the persistence of CP [152].

Several psychological interventions have shown promise in CP management, each targeting different aspects of the patient’s experience [152]. Commonly used therapies include

- Operant-behavioral therapy: Focuses on modifying behaviors that reinforce the pain experience [153].
- Cognitive behavioral therapy (CBT): Helps patients reframe negative thoughts about pain and develop coping strategies [154].
- Mindfulness-based stress reduction (MBSR): Uses mindfulness techniques to reduce stress and promote emotional well-being [155].
- Acceptance and commitment therapy (ACT): Encourages patients to accept pain and commit to living a meaningful life despite it [155].

These therapies have been shown to reduce pain-related distress and improve overall functioning, yet they remain underutilized in clinical practice [156].

It is crucial for healthcare providers to clearly communicate the role of psychotherapeutic intervention in CP management. Patients often misconstrue psychological support as an implication that their pain is psychosomatic or “in their head”. Clear communication can prevent these misunderstandings, encouraging patients’ engagement with psychological therapy and ultimately improving their ability to manage pain.

The panel of experts suggests that sex-aware psychological strategies, such as CBT and MBSR, should be incorporated, recognizing differences in emotional processing and coping mechanisms across the sexes (Table 2).

## 4. Conclusions

As the field of pain management evolves, there is growing recognition that relying on single-modality treatments is not an effective approach. In contrast, a holistic strategy that appropriately accounts for sex differences in CP is emerging as a more successful option.

A personalized approach to CP management is essential, given the profound influence of sex differences on pain prevalence, perception, and treatment responses. These differences necessitate that clinicians and researchers move beyond traditional biases and stereotypes in pain perception and adopt a more nuanced understanding of the complex interplay between biological, psychological, and social factors. Collaboration among specialists in pain medicine, psychology, and nursing enhances CP treatment effectiveness by addressing each aspect of the patient’s condition.

By acknowledging and addressing the biopsychosocial complexities of each patient, healthcare professionals can provide individualized, multimodal, and interdisciplinary care that better meets the distinct needs of both men and women.

## Figures and Tables

**Table 1:** Structured approach used to guide the literature selection and synthesis in each thematic section of the narrative review. * The cited references were identified either through the search terms specified in the table or by screening the reference lists of studies retrieved using those search terms.

Domain	Section	Search Terms Used	Cited References *
Epidemiological	3.1	“chronic pain” AND (“sex differences” OR “gender differences”) AND (“hormones” OR “prevalence” OR “epidemiology” OR “treatment satisfaction” OR “coping” OR “comorbidity”)	[4,6,7,8,9,10,11,12,13,14,15,16,17,22,23,24,28,29,48,49,50,51,52,53,54,55,56]
Biological mechanisms	3.2.1	“chronic pain” AND (“sex differences” OR “gender differences”) AND (“hormones” OR “hormonal fluctuations” OR “testosterone” OR “estrogen” OR “neuroimmune” OR “microglia” OR “T cells” OR “aging” OR “biomarkers”)	[15,16,17,28,29,30,31,32,33,49,50,57,58,59,60,61,62,63,64,65,66,67,68,69,70,71,72,73,74,75,76,77]
Psycho-social	3.2.2	“chronic pain” AND (“sex differences” OR “gender differences”) AND (“gender bias” OR “healthcare disparities” OR “social norms” OR “andronormativity” OR “cultural roles” OR “socio-economic status” OR “pain perception” OR “pain tolerance”OR “mental health” OR “depression” OR “anxiety” OR “coping strategies” OR “quality of life” OR “social support” OR “functioning” OR “pain acceptance”))	[6,16,17,23,24,39,40,41,42,43,44,45,46,78,79,80,81,82,83,84,85,86,87,88,89,90,91,92,93,94,95,96,97,98,99,100,101,102,103,104,105,106,107,108,109,110,111,112,113]
Pharmacological strategies	3.3.1	“chronic pain” AND (“sex differences” OR “gender differences”) AND (“analgesics” OR “NSAIDs” OR “ibuprofen” OR “opioid efficacy” OR “treatment response” OR “pharmacokinetics” OR “pharmacodynamics” OR “adverse drug reactions” OR “pharmacokinetics” OR “pharmacodynamics” OR “opioid metabolism” OR “drug clearance” OR “reproductive health” OR “endocrine effects”)	[34,37,38,114,115,116,117,118,119,120,121,122,123,124,125,126,127]
	3.3.2	“chronic pain” AND (“sex differences” OR “gender differences”) AND (“adverse drug reactions” OR “pharmacokinetics” OR “pharmacodynamics” OR “opioid metabolism” OR “drug clearance” OR “reproductive health” OR “endocrine effects”)	[39,128,129,130,131,132,133,134,135,136,137,138,139,140,141,142,143,144,145,146,147,148,149,150,151]
	3.3.3	“chronic pain” AND (“psychological interventions” OR “psychotherapy” OR “cognitive behavioral therapy” OR “mindfulness-based stress reduction” OR “acceptance and commitment therapy” OR “quality of life” OR “coping strategies”)	[152,153,154,155,156]

**Table 2:** Key take-home messages on sex differences in chronic pain. CP: chronic pain; NSAIDs: non-steroidal anti-inflammatory drugs.

Key Domains in CP Management	Sex-Specific Considerations and Strategies
Biological and psycho-social interplay	Sex-related differences in CP stem from a complex interaction of biological and psycho-social factors. Effective management requires tailored, interdisciplinary strategies that address both dimensions.
Pharmacological considerations	CP treatments, particularly opioids and NSAIDs, must account for sex-specific variations in drug response and adverse effects to optimize safety and efficacy.
Psychological interventions	The integration of psychological strategies enhances CP management outcomes. These interventions should be personalized according to sex and individual coping mechanisms.
Advancing precision medicine	Future approaches to pain management should prioritize gene therapy and the identification of sex-specific molecular targets to enhance precision and personalized treatment.
Equity and policy implications	Achieving equitable pain care necessitates awareness of sex differences in CP, addressing translational gaps, incorporating psycho-social factors, and considering sex-specific pharmacokinetics and therapeutic targets. Clinicians and policymakers are urged to advocate for sex-aware guidelines and individualized care pathways.

## Data Availability

No new data were created or analyzed in this study. Data sharing is not applicable to this article.

## Abbreviations

The following abbreviations are used in this manuscript:

ACT	Acceptance and commitment therapy
AE	Adverse effect
CBT	Cognitive behavioral therapy
COMT	Catechol-O-methyltransferase
CP	Chronic pain
HPA	Hypothalamic–pituitary–adrenal
IASP	International Association for the Study of Pain
MBSR	Mindfulness-based stress reduction
MOR	µ-opioid receptors
NIH	National Institutes of Health
NSAIDs	Non-steroidal anti-inflammatory drugs
OPRM1	Opioid receptor M 1
OXN	Oxycodone/naloxone
PCA	Patient-controlled analgesia
QoL	Quality of life
SES	Socio-economic status
